# FHIR Standard–Based Oncology Data Model for Cancer Screening: Design and Implementation Study

**DOI:** 10.2196/79011

**Published:** 2025-12-02

**Authors:** Manisha Mantri, Sayali Satokar, Pritam Tambe, Cheenmaya Bhutad

**Affiliations:** 1 Centre for Development of Advanced Computing (C-DAC), India Pune India

**Keywords:** cancer, screening, Fast Healthcare Interoperability Resources, FHIR, risk assessment, data model, oncology data model, ODM, interoperability

## Abstract

**Background:**

Cancer is a leading cause of death worldwide. Early detection through screening, diagnosis, and effective management can reduce cancer mortality. Risk assessment is crucial for improving outcomes by identifying high-risk individuals based on family history, genetics, lifestyle, and environment. Such targeted screening enhances accuracy and resource efficiency. However, the complex nature of oncology data—which includes clinical observations, lab results, radiology images, treatment regimens, and genetic information—presents significant challenges for data interoperability and exchange.

**Objective:**

This study proposes an oncology data model (ODM) based on the Fast Healthcare Interoperability Resources (FHIR) standard to facilitate the capturing, sharing, and processing of oncology data across various cancer care stages. We particularly focused on screening and risk assessment for 5 cancers: breast, cervical, esophageal, lung, and oral, within the Meghalaya Fourth Industrial Revolution for Sustainable Transformation Cancer Care pilot project in India.

**Methods:**

The ODM incorporates data elements from a cancer patient’s journey across 5 phases: encounter, risk assessment, clinical investigation, treatment, and outcome. Essential oncology data elements were modeled using the Health Level 7 FHIR Revision 4 standard. Custom FHIR profiles were developed for cancer-specific use cases, with terminology mapped to Systematized Nomenclature of Medicine–Clinical Terms, Logical Observation Identifiers Names and Codes, and the *International Classification of Diseases, 10th Revision*. The implementation guide (IG) was created using FHIR Shorthand, SUSHI Unshortens Short Hand Inputs, and the Health Level 7 IG Publisher. Technical and clinical validation and a stakeholder usability assessment were conducted using a demonstration tool designed for implementer training and adoption.

**Results:**

The data model enhances interoperability across the cancer care continuum, from screening to treatment. The resulting IG includes 25 oncology-specific resource profiles and 50 standardized terminology value sets that support both semantic and syntactic interoperability. Central to the model are the FHIR Questionnaire and QuestionnaireResponse resources, customized for structured data collection in clinical and community settings, supporting cancer screening workflows. Technical validation yielded FHIR conformance and terminology binding, while clinical validation by oncologists and public health experts confirmed the usability and relevance of 5 screening questionnaires. The demonstration tool promoted stakeholder engagement and practical evaluation of the FHIR profiles.

**Conclusions:**

The FHIR-based ODM offers a unified framework for structured, interoperable cancer data exchange from screening to after treatment. This study marks the first comprehensive Indian initiative to apply FHIR standards for oncology screening and risk assessment. Integrating with national digital health systems, like the Ayushman Bharat Digital Mission, can ensure consistent data sharing across screening programs, hospitals, and registries. Future work will focus on real-world model deployment, evaluation in multiple districts, expanding to treatment and survivorship data, and promoting national adoption to inform cancer policy, research, and precision oncology efforts.

## Introduction

Despite advances in treatment and early detection, the global cancer burden continues to rise due to factors such as population growth, aging, lifestyle changes, and environmental influences. Millions of new cancer cases and deaths occur each year, with lung, breast, colorectal, and prostate cancers being the most prevalent [1]. In India, cancer is a significant contributor to the burden of noncommunicable diseases, leading to substantial out-of-pocket expenditures for patients. In 2022, India recorded more than 1.46 million new cancer cases, resulting in a crude incidence rate of 100.4 per 100,000 individuals [2-4]. In the state of Meghalaya, India, there is a high prevalence of oral and esophageal cancers, primarily due to lifestyle factors, such as tobacco and betel nut consumption. The region’s unique sociocultural practices significantly contribute to these patterns. Access to cancer care facilities is limited, forcing many patients to seek treatment outside the state, which highlights the need for a robust framework for sharing data of patients with cancer to ensure continuity of care [4].

Cancer screening is a vital component of comprehensive cancer control, targeting early detection and timely intervention for cancer management. It offers significant benefits, including improved patient prognosis, reduced treatment costs, and enhanced public health outcomes. Unlike diagnostic procedures that are performed once symptoms appear, cancer screening targets individuals who are asymptomatic, enabling early identification of malignant neoplasm when they are most treatable and manageable. Recognizing this need, the Indian government has launched several targeted screening programs aimed at these prevalent cancers [5-7]. These initiatives strive not only to increase public awareness but also to improve access to screening services across the population [8]. To enhance the effectiveness of these programs, innovative technologies, such as artificial intelligence, machine learning, digital health platforms, mobile screening units, and cost-effective diagnostic methods, are being integrated into the screening process [9-11]. In addition, novel biomarkers specifically tailored to the Indian demographic are being explored to improve accuracy and facilitate early cancer detection. These efforts are being implemented through collaborative models involving central and state governments, nonprofit organizations, and private health care providers.

Cancer data are sourced from various platforms, such as screening systems, electronic health records, laboratory systems, imaging systems, and cancer registries. The lack of uniform data formats and protocols across these federated platforms results in incompatible data that are challenging to integrate, leading to delays in integration [12]. Incomplete data integration limits the effectiveness of clinical decision support systems, which rely on comprehensive data to provide actionable insights. Addressing these data interoperability issues requires concerted efforts [13,14]. Implementing standardized formats and protocols can greatly enhance the quality and efficiency of cancer screening, diagnosis, and treatment globally. The World Economic Forum (WEF), through the Center for the Fourth Industrial Revolution in India, has designed the Fourth Industrial Revolution for Sustainable Transformation (FIRST) in the health care sector. This strategy seeks to align all the stakeholders in the health care ecosystem and address critical challenges across 18 themes along 3 streams: prevention and early detection, curative care, and governance by using Fourth Industrial Revolution technologies. Cancer is the first theme that the FIRST Health portfolio will address, and the initiative has been named FIRST Cancer Care (FCC). The need for the development of an oncology data model (ODM) for interoperability and integration among screening applications, cancer health systems, diagnostic systems, cancer registries, and reporting platforms has emerged from the FCC report published by WEF [15].

The Fast Healthcare Interoperability Resources (FHIR) standard, developed by Health Level 7 (HL7) International, aims to standardize the representation and exchange of health care information [16]. FHIR’s compatibility with multiple data formats facilitates its implementation in diverse technological contexts. Using a flexible and robust framework such as FHIR in cancer data modeling aligns with the global and national efforts to harmonize health care data and improve clinical outcomes [17,18]. FHIR has been used in electronic health record data exchange, cancer survivor data sharing, and cancer clinical trials in multiple studies [19-22]. One notable initiative in oncology using the FHIR standard is the Minimal Common Oncology Data Elements (mCODE) to develop and maintain standard computable oncology data formats. mCODE is developed by HL7 Accelerator CodeX, a collaborative community that uses the FHIR standard to accelerate cancer data interoperability. However, mCODE only focuses on observations, conditions, status, stages, body structures (tumor), and genomic report, primarily during and after cancer diagnosis, with more than 37 profiles [23].

The Ministry of Health and Family Welfare, India, has adopted FHIR R4 as a base standard for data exchange under its Ayushman Bharat Digital Mission (ABDM), in which health systems connect through the ABDM Health Information Exchange for sharing the patient’s health records [14,24,25]. The FCC program pilot is implemented in the East Khasi Hills district of Meghalaya state in India. The FCC pilot project in Meghalaya concentrated on enhancing cancer screening awareness and conducting comprehensive screening in the East Khasi Hills district [26,27]. This focused approach aimed to bridge awareness gaps and ensure early detection and intervention at the community level. The execution of the project involved multiple project implementation partners working on a public-private cooperation model through a consortium, including the National Health Mission (NHM) Meghalaya as monitoring agency, Apollo Telemedicine Networking Foundation as field implementation and deployment, the National Programme for Prevention and Control of Non-Communicable Diseases as advisory, the WEF as coordination agency, and the Centre for Development of Advanced Computing as data model development agency. This paper particularly focuses on the design and development of the ODM, specifically oncology data modeling used for cancer screening using the FHIR framework [27-29]. ODM FHIR profiles and ValueSets were developed by adopting and mapping to existing ABDM profiles to support data interoperability and ease integration with other health systems in the country. ODM is the central component for interoperability between various cancer care applications.

## Methods

### Overview

**Figure 1:**
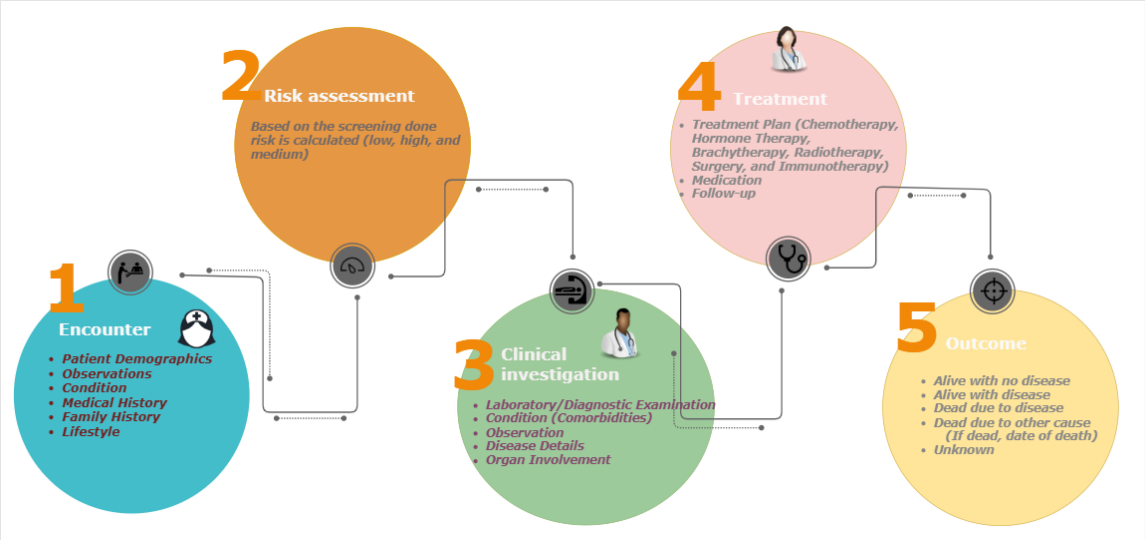
Patient journey.

The ODM is defined by considering the patient journey in cancer detection and treatment in 5 key phases, as illustrated in Figure 1.

#### Encounter

This is an initial phase involving the collection of patient information, including demographics, present conditions, medical history, family history, lifestyle, and test observations. Encounters may occur through screening programs, routine checkups, or incidental findings.

#### Risk Assessment

Risk assessment is performed based on the data collected during an encounter to classify the patient’s cancer risk as low, medium, or high. This step guides further investigation and care.

#### Clinical Investigation

Clinical investigation includes diagnostic examinations, clinical tests, and investigations aimed at confirming a cancer diagnosis. Data generated in this phase may be fragmented due to the complex nature of cancer care. For unconfirmed diagnoses, follow-up with routine monitoring is advised. A confirmed diagnosis moves the patient to treatment planning.

#### Treatment

Upon confirmation of cancer, a tailored treatment plan is developed based on the diagnosis. Treatment options include chemotherapy, hormone therapy, radiotherapy, surgery, immunotherapy, and follow-up care. These options are determined by factors such as disease stage, individual health, and specific cancer characteristics.

#### Outcome

The outcomes are assessed after the treatment. Possible outcomes include being alive without disease, alive with disease, deceased due to cancer, deceased due to another cause, or unknown status.

This structured journey emphasizes continuous data capture and a multidisciplinary approach to ensure comprehensive care for patients with cancer.

### FHIR Overview

**Figure 2:**
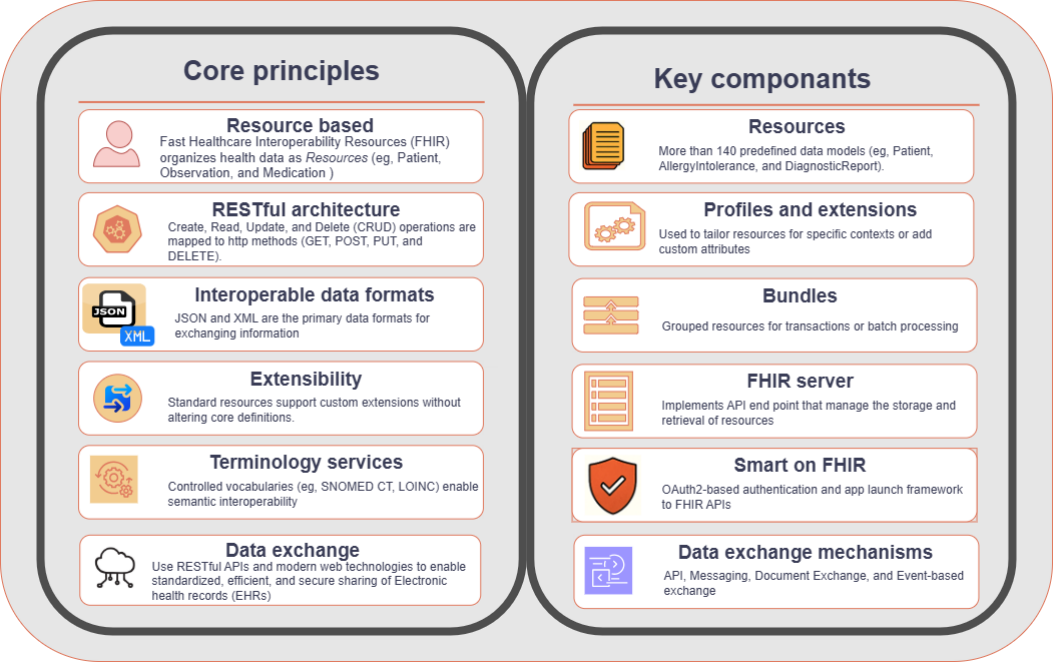
Fast Healthcare Interoperability Resources (FHIR) technical overview. API: application programming interface; CRUD: basic operations performed on the data: create, read, update, and delete; EHR: electronic health record; LOINC: Logical Observation Identifiers Names and Codes; OAuth2: open authorization protocol version 2.0; SNOMED CT: Systematized Nomenclature of Medicine–Clinical Terms.

FHIR is a modern health data exchange framework developed by HL7 International to enhance health care data interoperability. It adopts contemporary web technologies, particularly RESTful interfaces [30]. RESTful interfaces, also known as RESTful application programming interfaces (APIs), are a type of API that adheres to the architectural style of Representational State Transfer. The FHIR framework is structured around the modular components that represent various health care entities such as patients, medications, conditions, and observations, called *resources*, making it both human readable and implementable [31]. Its modular design is instrumental in managing complex datasets, such as datasets used in cancer research and treatment, including screening, genetic information, clinical trials, and treatment histories [32,33]. The normative status of FHIR R4 has enabled its use in many national health programs and health systems [19]. FHIR also enables customizing and extending the resources for a particular use case. These customizations are called profiles and extensions. A profile specifies additional rules and constraints on a base FHIR resource, such as making certain data elements mandatory, defining specific data types, or restricting allowed values for a field [34]. This framework aims to simplify data exchange and improve the usability of health care information systems through various exchange mechanisms and bundling methods. Figure 2 shows the core principles, components, and data exchange mechanisms supported by the framework.

The FHIR standard provides a framework for the seamless exchange of health care information electronically using modern web technologies, such as RESTful APIs and multiple data formats, including JSON, XML, and RDF. This standardized approach enables interoperability between health care systems for data sharing across platforms and devices. The core strength of FHIR lies in its fundamental building blocks—resources. Each resource is an entity that represents a specific type of health data. These modular units are designed for clarity and ease of data handling, enabling developers to create applications that seamlessly exchange and use these data [35]. For instance, patient demographics are recorded in the Patient resource, medical observations in the Observation resource, and family history in the FamilyMemberHistory resource.

### Development of Standard ODM

The lack of standardization in health care data leads to various organizations using diverse formats and systems for data collection and storage. This diversity can create challenges in sharing and integrating data across institutions, which may affect effective communication, collaboration, and the overall quality of patient care. A unified data model offers a compelling solution to this challenge. To tackle these challenges, leveraging emerging technologies is essential.

The FCC report, developed by stakeholders from various sectors, outlines a strategy for transforming cancer care. The Meg Can Care—Meghalaya Cancer Care program, the first of its kind state initiative in India, aims to enhance detection and treatment adherence using emerging technologies through public-private cooperation. These technologies, including smart screening devices, screening applications, and electronic medical records and hospital management information system solutions, must interoperate with each other for data exchange and continued patient care. By establishing standardized data capture processes, such a model would ensure consistency and compatibility across institutions. This, in turn, would facilitate seamless data sharing, leading to several key benefits. Enhanced communication and collaboration among health care providers would lead to more informed decision-making and, ultimately, better patient care. Standardized data would enable researchers to conduct large-scale studies more efficiently, leading to advancements in cancer prevention, diagnosis, and treatment. A unified data model would foster a more collaborative approach to cancer management across various health care providers, facilitating a more comprehensive approach to prevention, diagnosis, and treatment. The implementation of a standardized data model would extend beyond streamlining data exchange. Capturing data through targeted questions during cancer screenings provides a wealth of critical insights into various patient aspects, including demographics, present medical conditions, lifestyle choices, and family history. These details can be structured using specific FHIR resources, facilitating standardized data capture.

**Figure 3:**
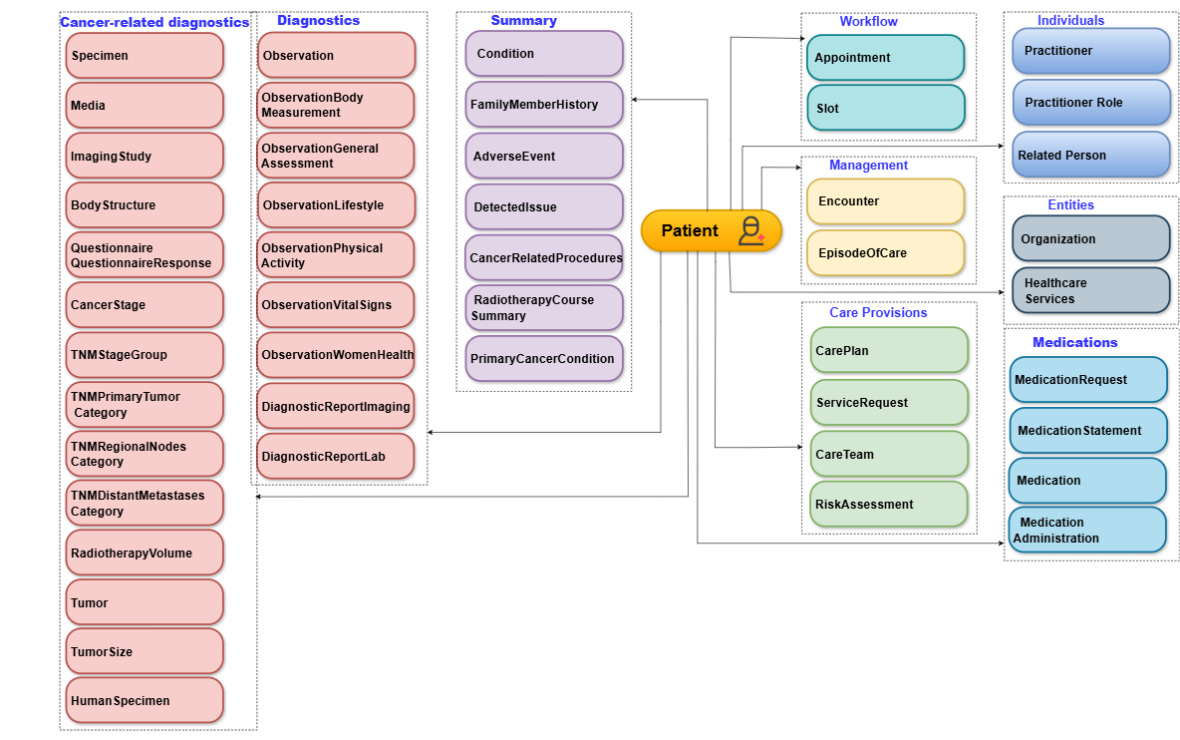
Oncology data model.

Figure 3 provides an overview of various classes referred to in the ODM. Different resources are used to create profiles for cancer-specific data throughout the phases of a patient’s cancer journey. Generic resource classes, such as patient, condition, medication, and outpatient department note, are directly derived as is or with additional customizations to accommodate cancer-specific data from ABDM.

### FHIR Profiles for ODM

#### Overview

The ODM aims to capture, represent, and exchange oncology-related data in a consistent format across health care systems. To achieve syntactic as well as semantic interoperability, ODM development involved 4 stages: data modeling, FHIR profiles development, terminology mapping, and FHIR implementation guide (IG) development.

#### Data Modeling

This foundational stage involves identifying the essential oncology data elements, data structures, and use cases and modeling these data structures in alignment with FHIR’s resource-based framework. It also entails identifying the basic resources and requirements of advanced or derivative resources for cancer-related data. A thorough analysis of clinical workflows and data sources in oncology care helped identify the essential data elements and their relationships. The selection of data elements occurred through a systematic and iterative process. This involved an in-depth review and analysis of Apollo Telemedicine Networking Foundation Cancer Screening and National Programme for Prevention and Control of Non-Communicable Diseases applications (mobile or tablet and portal) [28,36], community-based assessment checklist forms [37], and noncommunicable disease operational guidelines [38], followed by the preparation of the comprehensive reference documents.

#### FHIR Profile Development

Once core resources are identified, FHIR profiles are developed to tailor these resources to oncology-specific needs. Profiles specialize existing resources to capture additional data elements that are unique to oncology. For instance, a CancerDiagnosis profile might extend the *Condition* resource to capture specific details about cancer types and stages [39].

#### Terminology Mapping

Terminology mapping ensures semantic interoperability in oncology data. The data elements and value sets received from the existing state health programs and survey forms are mapped to standardized terminologies, including Systematized Nomenclature of Medicine–Clinical Terms (SNOMED CT), Logical Observation Identifiers Names and Codes (LOINC), and *International Classification of Diseases, 10th Revision*. A total of 50 standard terminology value sets were developed for precise data representation and mapped to the relevant codable data elements in FHIR resources. All the observation values were fully mapped with the terminology bindings [40].

#### FHIR IG Overview

The FHIR IG provides a detailed set of specifications and guidelines to address specific health care scenarios or use cases [41]. It ensures consistent implementation, interoperability, and compliance with established data-sharing frameworks. The FHIR IG is hosted in Meg Can Care for adoption and integration [42].

The FHIR IG and profiles for ODM are developed based on FHIR R4 and refer to ABDM profiles using the following tools:

- FHIR Shorthand (FSH) is a specially designed language for defining the content of the HL7 FHIR IG. It is designed to be simple and compact and, along with SUSHI Unshortens Short Hand Inputs (SUSHI), can be used to produce FHIR profiles, extensions, and IGs [43].
- SUSHI is an FSH compiler. SUSHI converts FSH language to FHIR artifacts. SUSHI can run in stand-alone mode or as part of the HL7 IG Publisher [44].
- FHIR Validator is a Java command-line tool to validate FHIR profile instances [45]. The definitions of the profiles were created based on the FHIR specification in FHIR (version R4 4.0.1).
- FHIR IG Publisher is a tool to generate, validate, and publish FHIR IGs. It produces a static website for the guide, which can be hosted on web servers [46].

### Ethical Considerations

The research reported in this manuscript does not involve human participants or animal subjects; ethical approval was not applicable.

## Results

A structured set of standardized FHIR profiles was developed to support the representation and exchange of oncology-related data, with a focus on enhancing syntactic and semantic interoperability across the cancer care continuum. The implementation effort culminated in the publication of a dedicated FHIR IG. The guide comprises 25 distinct resource profiles tailored to cancer screening, assessment, and treatment workflows. In addition to the resource profiles, a total of 50 standardized terminology value sets were curated to ensure consistency in data capture and coding. These value sets facilitated alignment with recognized terminologies and ontologies, supporting uniform interpretation and reuse of data across systems. This work aimed to minimize the use of extensions by reusing existing data elements from the base standard. However, based on the requirements, 7 extensions related to radiotherapy were developed and used in a controlled manner to ensure flexibility without compromising interoperability. Furthermore, to facilitate training and stakeholder engagement and promote adoption of the ODM, a demonstration application was developed. This application served as a practical tool to showcase the implementation of the FHIR profiles, enabling hands-on exploration and feedback from end users and decision-makers.

Key resources used in oncology data modeling were the FHIR Questionnaire and QuestionnaireResponse resources. Custom profiles were developed for both, reflecting the critical role of cancer screening in early detection and care coordination. These profiles were specifically designed to support the interoperable collection of structured oncology data from clinical environments and community-based digital health platforms. The Questionnaire profile includes 2 mandatory elements: the title and its intended purpose. The profile necessitates the inclusion of at least 1 item within the questionnaire. The data mapping process involves creating an instance of the questionnaire profile populated with actual information.

**Figure 4:**
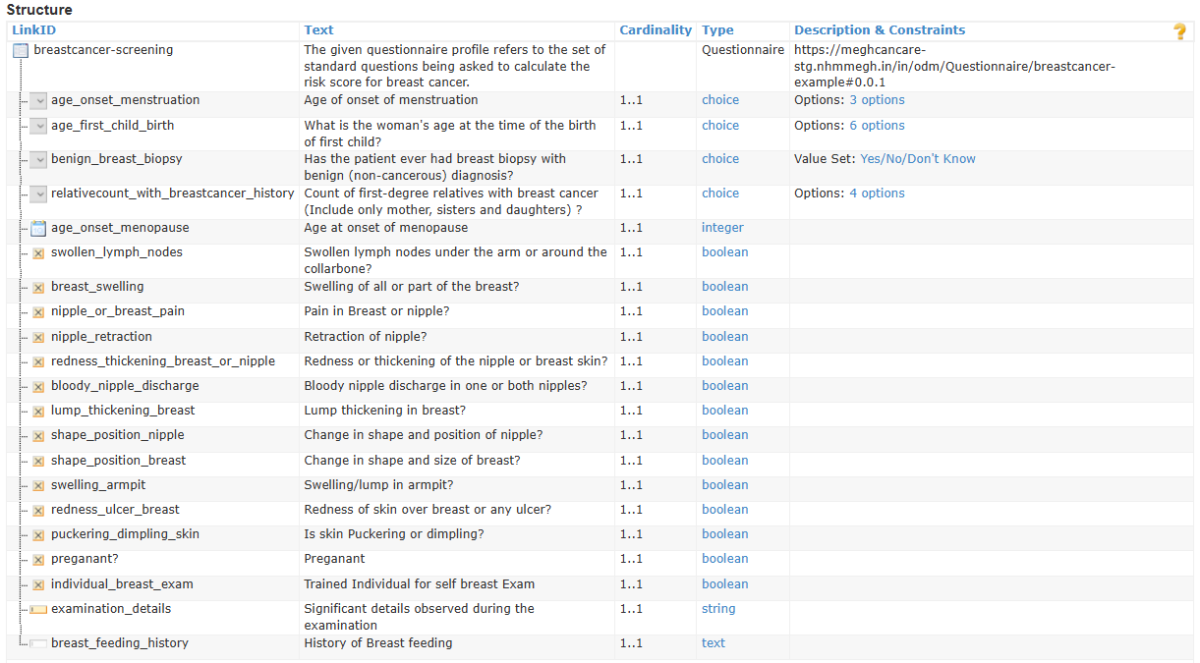
Questionnaire—breast cancer.

The cancer screening questionnaires included 21 questions for breast cancer, 20 questions for cervical cancer, 18 questions for esophageal cancer, 20 questions for lung cancer, and 19 questions for oral cancer. These questions were collated from stakeholder applications and validated by practitioners in NHM Meghalaya. Implementers can assign weights to the responses to define cancer risk, which can then support further treatment planning and analysis. On the basis of the Questionnaire profile, 5 separate instances were created for all 5 types of cancers. These instances were developed based on inputs from the existing cancer programs in the state and through multiple stakeholder consultations, including oncology specialists at NHM Meghalaya. The instance of the Questionnaire resource with the standard question set to calculate the risk score for breast cancer is shown in Figure 4. A similar standard questionnaire set was created for cervical, esophageal, lung, and oral cancers.

**Figure 5:**
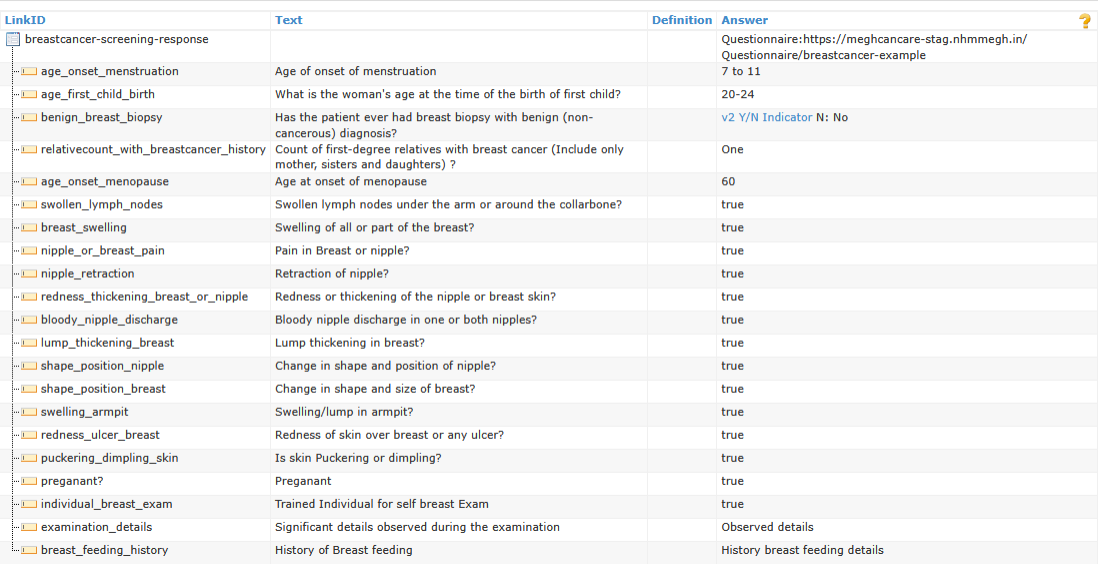
Breast cancer questionnaire response.

**Figure 6:**
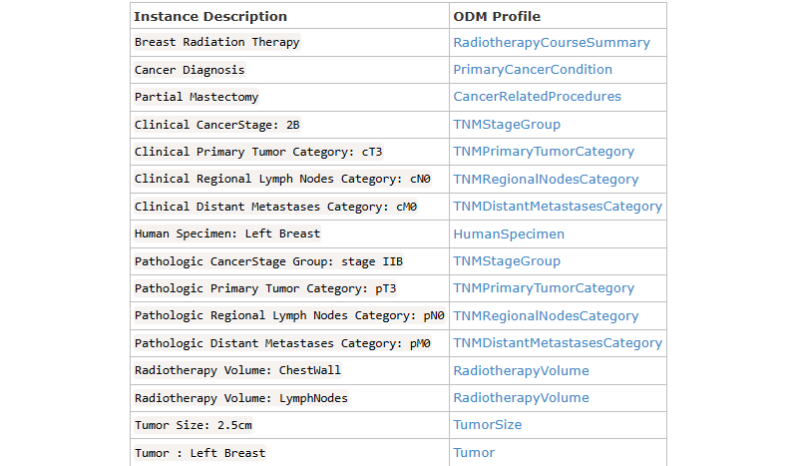
Fast Healthcare Interoperability Resources–based instances vis-à-vis oncology data model (ODM) profiles.

Each question in the questionnaire is associated with the *LinkId* element to uniquely identify the question. The *Text* element stores the text of a question. The *Cardinality* defines whether the mentioned question must be answered or not, and *Type* shows the data type of the expected answer. The QuestionnaireResponse profile facilitates the capture of diverse answers, with the values in Boolean, numerical data, paragraph-length text entries, single-choice selections, and multiple-choice selections. Responses are stored within a designated QuestionnaireResponse profile instance. The LinkId element value assigned to each question enables the mapping between individual responses and their corresponding questions within the template. Figure 5 illustrates the QuestionnaireResponse Profile instance for breast cancer. Similarly, a few more data instances generated out of the ODM profiles are shown in Figure 6. Data captured from screening examinations for cancers served as the core input for risk assessments to evaluate the likelihood of cancer development. The FHIR RiskAssessment profile was adopted for the result representation. This profile covers patient information, condition, observations, diagnostic reports, documents from the ABDM FHIR profiles, and the *Prediction* element to capture the assessment’s result. The prediction can be expressed either in textual form or via a standardized terminology code, such as SNOMED CT.

Technical validation focused on assessing syntactic correctness, conformance to FHIR specifications, and interoperability performance of the ODM. The validation process was carried out using the HL7 FHIR Validator and the HL7 Application Programming Interface–FHIR Java command-line tool for all 25 oncology-specific FHIR resource profiles and 50 value sets. All the resource instances passed syntactic validation against the ODM IG with a successful mapping of coded data elements to SNOMED CT; LOINC; or *International Classification of Diseases, 10th Revision* concepts, confirming strong semantic alignment. The clinical validation was designed to ensure clinical completeness, usability, and relevance of the data elements and screening workflows. The 5 cancer-specific screening questionnaires were reviewed by domain experts, including oncologists, public health specialists, and field implementers from the Meghalaya Cancer Care Program. Stakeholder engagement and usability assessments were conducted using the demonstration tool to evaluate readiness for implementation within the FCC ecosystem.

## Discussion

The larger scope of ODM is to establish a unified information model that integrates seamlessly with all cancer care applications, including screening, diagnosis, treatment, postoperative care, and the cancer registry. This integrated approach helps in enhancing the comprehensiveness and utility of the data for effective disease surveillance, policymaking, and research.

The profiles and the questionnaire template developed as part of the project have been developed, and efforts were undertaken to ensure the template is tailored to the specific demographic characteristics and needs. We have incorporated a mechanism for periodic revision and updates of the questionnaire template. This revision process is crucial for capturing accurate and detailed screening information, enabling the model to address changes in demographic profiles, emerging challenges, and evolving needs. Currently, the ODM is under review and in the integration stage, and it requires thorough consultation with domain experts as well as validation from all relevant stakeholders. To the best of our knowledge, this initiative represents the first comprehensive work in India to develop and implement an ODM for cancer screening and implement Questionnaire and Questionnaire Response resources using international standards, such as FHIR, SNOMED CT [47], LOINC [48], and TNM staging—a classification system for malignancy [49]. This positions the ODM as an early initiative in oncology informatics.

Development of ODM required a continuous harmonization among diverse stakeholders to ensure consensus on the essential oncology data elements. Despite encouraging validation outcomes, several challenges were identified during development and evaluation. FHIR standard literacy among the stakeholders was one of the key challenges, which was resolved through a series of internal workshops on the standard, FHIR resources, value sets, and use of the IG, and so on. Future adoption may also be constrained by limited familiarity with FHIR standards among health IT implementers. The current testing remains limited to simulated data instances. A real-world validation using live screening data in the next phase of the project will provide more insights and strengthen the ODM conformance. The existing electronic medical records and registries are not yet FHIR enabled and require bridging mechanisms for interoperability.

The ODM’s (although originally developed for the Meg Can Care project) use of international standards facilitates its adoption by implementers globally. National-level adoption can be beneficial to address the challenges of data collection and coding [50]. However, such a larger adoption requires harmonization of these datasets to standardized terminologies. In addition, national-level adoption requires a live implementation showcase at the district and state levels, central endorsement, and inclusion under the ABDM framework for its scalability and consistent data sharing across screening programs, hospitals, and registries. The ODM offers a framework for integrating cancer screening programs, cancer hospitals, and cancer registries into an interoperable cancer network. By leveraging FHIR’s RESTful APIs, the model can support efficient data exchange and seamless integration with health care systems. Future work shall focus on real-world deployment and model evaluation in multiple districts; expansion to treatment and survivorship data; and national adoption to inform cancer policy, research, and precision oncology efforts. This expansion would offer broader benefits to researchers and the cancer care community. While similar models exist in regions such as mCODE, a globally harmonized framework could address oncology data requirements across diverse health care systems, fostering international collaboration and advancing cancer research and care worldwide.

## Abbreviations

ABDM: Ayushman Bharat Digital Mission
API: application programming interface
FCC: Fourth Industrial Revolution for Sustainable Transformation Cancer Care
FHIR: Fast Healthcare Interoperability Resources
FIRST: Fourth Industrial Revolution for Sustainable Transformation
FSH: Fast Healthcare Interoperability Resources Shorthand
HL7: Health Level 7
IG: implementation guide
LOINC: Logical Observation Identifiers Names and Codes
mCODE: Minimal Common Oncology Data Elements
NHM: National Health Mission
ODM: oncology data model
SNOMED CT: Systematized Nomenclature of Medicine–Clinical Terms
SUSHI: SUSHI Unshortens Short Hand Inputs
WEF: World Economic Forum

## Data Availability

Data sharing is not applicable to this paper as no datasets were generated or analyzed during this study.
